# A Small Protein but with Diverse Roles: A Review of EsxA in Mycobacterium–Host Interaction

**DOI:** 10.3390/cells10071645

**Published:** 2021-06-30

**Authors:** Yanqing Bao, Lin Wang, Jianjun Sun

**Affiliations:** Department of Biological Sciences, Border Biomedical Research Center, University of Texas at El Paso, 500 West University Avenue, El Paso, TX 79968, USA; ybao@utep.edu (Y.B.); lwang4@utep.edu (L.W.)

**Keywords:** *Mycobacterium tuberculosis*, EsxA, membrane-permeabilizing activity, immunoregulator, host–pathogen interaction

## Abstract

As a major effector of the ESX-1 secretion system, EsxA is essential for the virulence of pathogenic mycobacteria, such as *Mycobacterium tuberculosis* (Mtb) and *Mycobacterium marinum* (Mm). EsxA possesses an acidic pH-dependent membrane permeabilizing activity and plays an essential role by mediating mycobacterial escape from the phagosome and translocation to the cytosol for intracellular replication. Moreover, EsxA regulates host immune responses as a potent T-cell antigen and a strong immunoregulator. EsxA interacts with multiple cellular proteins and stimulates several signal pathways, such as necrosis, apoptosis, autophagy, and antigen presentation. Interestingly, there is a co-dependency in the expression and secretion of EsxA and other mycobacterial factors, which greatly increases the complexity of dissecting the precise roles of EsxA and other factors in mycobacterium–host interaction. In this review, we summarize the current understandings of the roles and functions of EsxA in mycobacterial infection and discuss the challenges and future directions.

## 1. Introduction

**Figure 1:**
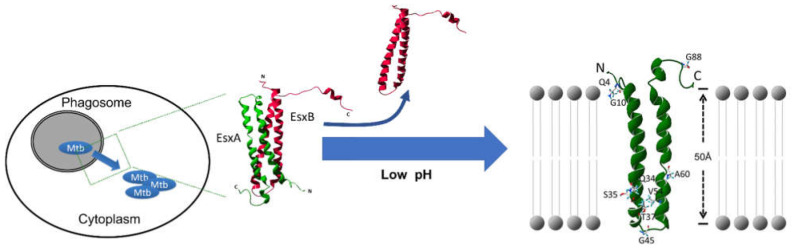
The model of EsxA-mediated Mtb cytosolic translocation. Mtb is internalized into the phagosome of macrophage. Upon acidification, the secreted EsxAB heterodimer is dissociated, and EsxA inserts into the membrane, which facilitates Mtb escape from the phagosome and translocation into the cytosol for intracellular replication. The figure is modified from [61].

*Mycobacterium* is a genus that consists of at least 188 different species [1,2,3,4]. Several of them, especially *Mycobacterium tuberculosis* (Mtb), are imposing a great threat to public health due to their strong virulence and pathogenicity to humans [5,6,7,8,9,10,11,12]. As the causative pathogen of tuberculosis disease (TB), Mtb infects around one-third of the world population and causes 1.4 million deaths each year worldwide [13]. The global TB epidemic is largely attributed to the ability of Mtb to invade and stay persistent in host cells [14,15,16,17,18,19]. Hundreds of genes necessary for Mtb intracellular survival have been identified [6,20,21,22,23,24,25]. Among these genes, EsxA (6 kDa early secreted antigenic target, ESAT-6) and EsxB (10 kDa culture filtrate protein, CFP-10) have drawn much attention for their essential roles in Mtb virulence [26,27,28,29]. The *esxBA* operon is located within the *esx-1* locus that encodes the ESX-1 Type VII secretion system [30,31,32,33]. Both EsxA and EsxB are found in the Mtb culture supernatant and considered as the secreted substrates of the ESX-1 system [34]. EsxA was first identified as a potent T-cell antigen [35,36]. Therefore, it has become a major target of new vaccine development against Mtb [37,38,39,40]. EsxA is upregulated during Mtb infection [41], and gene deletion or the impaired secretion of EsxA leads to reduction in Mtb intracellular survival [28,29,30,42], which implicates EsxA as playing an essential role in Mtb virulence. EsxA mediates mycobacterial escape from the phagosome and translocation to the cytosol (here termed cytosolic translocation) through its acidic-pH-dependent membrane-permeabilizing activity (MPA) that disrupts lipid membranes at low pH (Figure 1) [43,44,45,46,47,48,49,50,51,52]. Interestingly, other mycobacterial factors are also involved in mycobacterial cytosolic translocation, and the co-dependent expression and secretion of EsxA with other factors increases the complexity of dissecting the precise roles of EsxA in mycobacterial pathogenesis [43,53,54,55]. Moreover, EsxA is a strong immunoregulator and interacts with multiple cellular proteins and signaling pathways [35,36,40,56,57,58,59,60]. In this review, we summarize the current understandings of the diverse roles of EsxA in mycobacterial pathogenesis and regulation of host immune responses, which is followed by a discussion of the challenges and future directions.

## 2. Current Understanding of EsxA’s Role in Mycobacterium–Host Interaction

### 2.1. The EsxA MPA Mediates Mycobacterial Cytosolic Translocation

BCG (Bacillus Calmette–Guérin), an attenuated strain of *Mycobacterium bovis,* has been used as a TB vaccine for one hundred years. Genomic comparison between Mtb and BCG has found that the region of difference 1 (RD1), which is present in Mtb but absent in BCG, is essential for mycobacterial virulence [28,30,62,63,64,65,66]. RD1 is located in the *esx-1* locus and includes the *esxBA* operon. The deletion of RD1/ESX-1 attenuates mycobacterial virulence (e.g., Mtb and Mm) [28,53] and specifically reduces their ability to penetrate the phagosome and translocate into the cytosol for replication and cell-to-cell spreading, which is a major mechanism of mycobacterial virulence (Figure 1) [47,48,52]. Deletion of the *esxBA* operon or impairment of the secretion of EsxA and/or EsxB cause an equivalent virulence attenuation compared to the deletion of RD1, indicating that EsxA and EsxB are the virulence effectors of the ESX-1 system [28,43,49,50].

Biochemical studies demonstrate that EsxA protein, either purified from *Escherichia coli* (*E. coli*) or Mtb culture supernatant, possesses an acidic pH-dependent MPA [46,67]. Although the homologues of EsxA exist across different mycobacterial species [68,69,70], such activity is only found in the EsxA proteins from pathogenic mycobacteria, such as Mtb and Mm [46], but not from non-pathogenic *Mycobacerium smegmatis* (Ms), which links the EsxA MPA with mycobacterial virulence. Upon acidification, EsxA undergoes significant conformational changes and disrupts liposome membranes [46,67]. The central helix-turn-helix motif of EsxA inserts into the liposome membrane to form a membrane-spanning structure, while the N- and C-terminal flexible arms hang on the surface of the membrane (Figure 1) [46,61]. Site-directed mutagenesis has found that Gln5 (Q5) is the critical residue for the MPA. Replacing Q5 with a basic residue (e.g., Q5K and Q5R) diminishes the MPA, while replacing it with a hydrophobic residue (e.g., Q5V, Q5I) enhances the MPA. Importantly, the virulence of the Mtb/Mm strains carrying the mutations Q5K or Q5V is either attenuated by Q5K or enhanced by Q5V, which establishes a clear link between the EsxA MPA and the ability of mycobacteria to penetrate the phagosome and translocate into the cytosol for replication and cell-to-cell spreading [44]. Most recently, a 13-amino-acid SpyTag (ST) was engineered into the C-terminus of EsxA without significant impact on the expression, secretion, and MPA of EsxA. The EsxA-ST can only be specifically recognized and conjugated by the SpyCatcher (SC)-GFP through the covalent bonding between ST and SC after EsxA-ST is secreted out of mycobacteria. Thus, the SC-GFP binding to EsxA-ST only inhibits the EsxA MPA and has no effect on other co-dependently secreted factors. Inhibition of the EsxA-ST MPA at the post-secretion level by SC-GFP significantly reduces the Mm intracellular survival, which confirms the essential role of the EsxA MPA in mycobacterial virulence [71].

The MPA is present in the EsxA protein from pathogenic mycobacteria, such as Mtb and Mm, but absent in the orthologous EsxA protein from non-pathogenic Ms [46,72]. Earlier studies have shown that Ms also has a conserved and functional ESX-1 system that can secrete Mtb EsxA and EsxB [73], and this ESX-1 system is essential for DNA transfer in Ms [74], but the putative role of EsxA in non-pathogenic mycobacteria is not clear.

### 2.2. EsxA Disassociates from EsxB to Exhibit MPA

**Figure 2:**
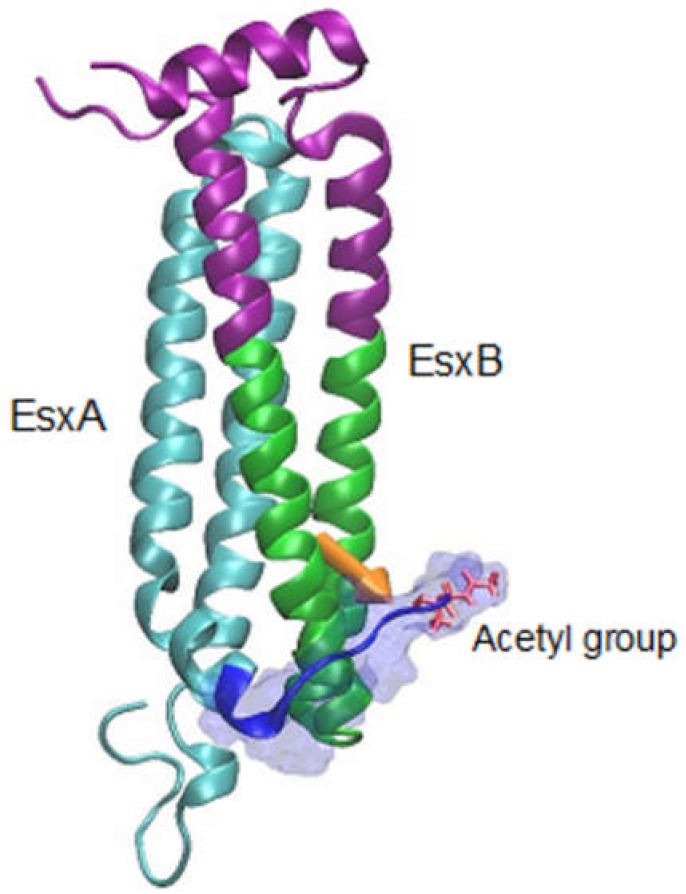
Interaction between the N^α^-acetylated EsxA and EsxB at pH 4. The structure of Mtb EsxAB heterodimer with N^α^-acetylation was analyzed by molecular dynamic simulation. The figure was generated from snapshots of 20 ns molecular dynamic (MD) simulations at pH 4. EsxA is colored in cyan; the N-terminus of EsxA is colored in blue and shown in transparent surface representation. The acetylated Thr-2 residue is shown in bond representation and colored in red. The residues in EsxB within 20 Å of the N-terminus of EsxA are colored in green, and the residues beyond 20 Å are shown in purple. The orange arrows represent the electrostatic interaction between two sets of residues: the residues in the N-terminus of EsxA (blue) and the residues in EsxB within 20 Å of the N-terminus of EsxA (green). The MD simulation shows that the acetylated N-terminal arm of EsxA makes direct contracts with EsxB in a frequent “bind-and-release” mode, which generates a force of 44 pN to pull EsxB away from EsxA. The figure is modified from [86].

EsxA and EsxB are expressed from the *esxBA* operon, form a heterodimer (here termed EsxAB) inside mycobacteria, and secreted out of mycobacteria in a co-dependent manner [27,75,76,77,78,79]. EsxAB exhibits structural changes and biological functions, such as binding to macrophages, regulation of NF-κB transactivation, and inhibition of autophagy [27,78,80,81]. An earlier study with the native EsxA and EsxB proteins isolated from the culture filtrate has proposed a model in which the EsxAB is dissociated upon acidification, allowing EsxA to interact with lipid membranes [67]. However, the recombinant EsxA (rEsxA) purified from *E. coli* remains binding to EsxB even at a low pH condition [79]. This has intrigued a hypothesis that the EsxA protein produced in mycobacteria has unique post-translational modifications that are not available in *E. coli* [82]. Indeed, EsxA produced in mycobacteria is acetylated at the Thr2 residue after Met1 is removed, which is a process called N^α^-acetylation. Furthermore, EsxB preferably binds to the nonacetylated EsxA than the acetylated EsxA, indicating that N^α^-acetylation at Thr2 is the key factor facilitating the dissociation of EsxAB at low pH [83,84]. Moreover, the homeostasis of N^α^-acetylation of EsxA is correlated to the mycobacterial virulence [85]. A recent study with site-directed mutagenesis at Thr2 has obtained direct evidence that the mutations at Thr2, which precludes N^α^-acetylation, inhibits the heterodimer separation and hence prevents EsxA from interacting with the host membrane, resulting in attenuated mycobacterial cytosolic translocation and virulence. Interestingly, molecular dynamics (MD) simulations have revealed the molecular mechanism underlying the heterodimer dissociation. The MD simulations show that at low pH, the N^α^-acetylated Thr2 makes direct and frequent “bind-and-release” contacts with EsxB, which generates a force that pulls EsxB away from EsxA (Figure 2) [86]. N^α^-acetylation of EsxA at Thr2 is a potential target for anti-TB drug developments.

### 2.3. EsxA Is a Strong Immune Regulator

As a strong immunoregulator, EsxA interacts with several host proteins and is involved in a number of cellular immune pathways. The purified EsxA binds with the immobilized laminin protein in a dose-dependent manner. Laminin is enriched in the membranes of human alveolar epithelial cells A549 and WI-26 and is believed to mediate the binding between EsxA and the cells [59]. In the airway tract, Mtb entry into epithelial M cells requires a functional ESX-1 system. The beads coated with the purified EsxA efficiently translocate across the M cell layer, suggesting that EsxA plays a role in mediating Mtb entry into the M cells. Subsequent co-immunoprecipitation identifies that EsxA interacts with scavenger B-1 in M cells, indicating that scavenger B-1 may function as a surface receptor for EsxA to mediate Mtb invasion in cells and animal models [60].

In macrophages, EsxA and EsxAB bind to beta-2-microglobulin (β2M) through the C-terminus of EsxA. When incubated with THP-1 cells, EsxA and EsxAB are translocated into endoplasmic reticulum (ER), where β2M forms the MHC-I complex. Following the incubation, the cell membrane association of β2M and the peptide presentation of MHC-1 are decreased, indicating that EsxA inhibits macrophage antigen presentation by interacting with β2M [58]. EsxA binds to the cell surface of RAW264.7 cells in a TLR2-dependent manner. The ELISA assay conducted with the purified EsxA or the EsxA-derived peptides demonstrates that the C-terminus of EsxA directly binds to TLR2 extracellular domain and attenuates the responses of RAW264.7 cell to TLR2 ligand stimulation. Further experiments with inhibitors or genetic silencing have confirmed that PI(3)K and Akt mediate the attenuation effect [57]. In murine dendritic cells, EsxA induces a strong production of IL-6 and TGF-β in a TLR-2-dependent manner, which directs Th17 differentiation for protective response against Mtb infection [87].

In addition to interacting with cellular proteins, EsxA stimulates immune responses as a potent T-cell antigen [35,36]. Most epitopes are located in the N-terminus of EsxA, and the residues 51–60 are effectively recognized by T cells [88]. Upon EsxA treatment, the peripheral blood monocyte cells (PBMCs) isolated from the Mtb-infected donors produce significant amounts of cytokines, including IFN-γ, IL-2, IL-6, IL-8, IL-10, MCP-1, MIP-1α, and TNF-α [35,89,90,91]. IFN-γ, IL-2, and TNF-α are mainly produced through Th1 response [92]. IFN-γ plays a central role for immunity against Mtb by activating macrophage to kill intracellular Mtb and recruiting immune cells to infection sites [93,94,95,96]. Hosts with deficiencies in IFN-γ or its corresponding receptor exhibit higher susceptibility and severity to Mtb infection [94,97,98,99]. As a pro-inflammatory cytokine, TNF-α induces the apoptosis of the alveolar macrophages infected with Mtb, which limits Mtb intracellular survival [100]. It coordinates with IFN-γ to induce reactive nitrogen intermediates against Mtb in macrophages [101,102]. By inducing the migration of immune cells to the infection sites, TNF-α is critical for the formation of Mtb-containing granuloma. Inhibition of TNF-α impairs granuloma formation and increases the severity of Mtb infection [103,104]. IL-2 mainly promotes T-cell proliferation to affect the magnitude of immune response [105]. Since the IL-2/IFN-γ secretion profile in the T cells infected with either active and or inactive Mtb is different, it is promising to apply IL-2/IFN-γ detection to discriminate active TB patients from healthy or vaccinated people [106,107,108,109].

IL-6 and IL-10 are the major immune regulators during Mtb infection. In the acute or early stage of infection, IL-6 is required for IFN-γ production and protective response against Mtb [110,111]. It is also required for Th1 immune responses induced by subunit vaccination with Mtb extraction [112]. In the cultured macrophages, IL-6 is negatively correlated with the activation of IFN activation pathways [113,114]. The inhibition of the IL-6 trans-signaling pathway yields therapeutic effects on inflammation caused by Mtb infection without significantly increasing bacterial burden, indicating that IL-6 plays a major role in immune regulation in TB progression [115]. IL-10 was firstly identified as an inhibitor of Th1 cell cytokines [116]. During Mtb infection, IL-10 inhibits the production of pro-inflammatory cytokines, which are responsible for macrophage antigen presentation. Thus, the subsequent T-cell activation is inhibited by IL-10 [117]. Deficiency in IL-10 enhances immune responses against Mtb infection and reduces the bacterial burden in mouse lung [118]. For innate killing against Mtb, IL-10 blocks maturation of the Mtb-containing phagosomes and enhances Mtb intracellular survival in macrophages [119]. In the Mtb-infected macrophages, IL-10 counteracts the TNF-α-induced apoptosis and reduces nitric oxide production as well as caspase-1 activation [120].

IL-8, MCP-1, and MIP-1α are chemokines responsible for cell migration and granuloma formation, which are parts of immune responses induced by Mtb infection [121,122,123,124]. During Mtb infection, IL-8 is secreted by alveolar macrophages, peripheral blood lymphocytes, and alveolar epithelial cells upon Mtb infection [125,126,127]. It mainly stimulates the migration of neutrophils and lymphocytes during Mtb infection [128,129]. IL-8 is significantly upregulated in the infected-tissue fluid and the neutrophil-infiltrated granuloma in TB patients [129,130,131]. The neutralization of IL-8 reduces granuloma formation in animal models [132]. MCP-1 is secreted by macrophages upon Mtb infection or TNF-α stimulation [133,134]. Similar to IL-8, the production of MCP-1 is significantly upregulated in pleural effusion of TB patients [129], and it increases macrophage recruitment to lung [135]. MCP-1 also functions as an inhibitor of the immune responses against Mtb. Genetic studies demonstrate that populations with certain MCP-1 gene mutations produce high concentrations of MCP-1, which inhibits IL-12p40-mediated immune responses against Mtb [136]. MIP-1α is secreted by neutrophils and macrophages upon Mtb infection [129,137]. Similar to MCP-1, MIP-1α is partially dependent on TNF-α stimulation [138]. In coordination with MCP-1, MIP-1α mediates the migration of neutrophils to granuloma [139]. It also stimulates Th1 response by driving T-cell differentiation and enhancing IFN-γ secretion [140,141].

In recent years, EsxA has also been found to modulate host immune responses by regulating host microRNAs (miRNA). By repressing miRNA let-7 in an EsxA-dependent manner, Mtb upregulates the expression of A20, an NF-κB inhibitor, and inhibits macrophage function, which significantly upregulates Mtb survival [142]. EsxA upregulates the miRNA155 transcription in macrophages, which inhibits the production of cyclooxygenase-2 and IL-2, hence upregulating apoptosis [143,144]. The upregulation of miRNA155 inhibits autophagy, which favors Mtb survival in mouse macrophages and human dendritic cells [143,145]. Either the purified EsxA or Mm infection downregulate miR-148 or miR-147 in mouse macrophages, which increases Mm intracellular survival, suggesting that EsxA increases Mm intracellular survival by inhibiting the two miRNAs [146,147].

### 2.4. EsxA-Mediated Cytotoxic Effects: Necrosis and Apoptosis

Mtb infection of macrophages leads to both necrosis and apoptosis [148,149,150], while Mtb infection of lung epithelial cells and neutrophils mainly leads to necrosis [151,152,153,154]. The mycobacterial strains without EsxA cause significantly lower cell death [49,155], suggesting that EsxA is essential for the cytotoxic effects.

Purified EsxA induces intracellular Ca^2+^ overload to activate calpain-dependent necrosis in aged neutrophils, which might be related to EsxA interaction with the externalized phosphatidylserine on neutrophil cell surface [153]. Mycobacterial infection upregulates macrophage necrosis and the secretion of IL-1β and IL-18 through the activation of inflammasome pathways [56,150,156,157,158]. The studies with genetic silencing methods further identified that EsxA activates the NLRP3/ASC complex [56,157]. By causing phagosome or lysosome leakage, EsxA induces the release of cathepsin B into the cytosol to activate NLRP3/ASC, which is critical for macrophage necrosis [158,159]. In lung epithelial cells, EsxA-mediated necrosis is closely related to mycobacterial virulence [151,155]. EsxA increases mycobacterial intracellular replication, which overwhelms lung epithelial cells by increased membrane permeation and necrosis [71,151,160]. In the epithelial cells incubated with EsxA, necrosis is upregulated through the activation of MAPK and ERK pathways [161].

The effects of EsxA on apoptosis have been widely studied. EsxA protein induces apoptosis in both epithelial and macrophage cells through various pathways. Incubation with the purified EsxA protein, but not other Mtb proteins, induces apoptosis in THP-1 cells, which is blocked by anti-EsxA antibody [162]. Since the transcription of caspase-1, -3, -5, -7, -8, and -9 are upregulated, it strongly indicates that EsxA induces apoptosis through caspase pathways [162,163]. As a regulator of ER stress response, BAT3 inhibits the EsxA-induced macrophage apoptosis, indicating that EsxA also mediates apoptosis through the ER stress response pathway [164]. In lung epithelial cells, EsxA induces apoptosis by upregulating the expression of caspase-3, -4, and -12 through the ER stress response [165]. The EsxA protein also regulates caspase-3 activation through the miR-155-SOCS1 pathway for apoptosis [144]. In infection assays, Mtb, but not the mutant strain without EsxA, upregulates Bcl-2-interacting mediator and induces macrophage apoptosis in vitro [166]. In vivo, Mtb induces macrophage apoptosis in granuloma, while the ΔRD1 strain exhibits a significantly lower ability to induce apoptosis [167]. All these studies have demonstrated that EsxA plays an essential role in host cell apoptosis.

### 2.5. Effects of EsxA on Autophagy

Autophagy is a fundamental cellular function to maintain the homeostasis of the intracellular environment [168], including the removal of intracellular pathogens [169]. When mycobacteria gain access to the cytosol, the rupture of mycobacteria-containing vacuole triggers the STING pathway to induce autophagy [170,171]. Followed by enhanced fusion with lysosome, autophagy selectively degrades mycobacteria to inhibit its intracellular survival [172,173]. As a Mtb virulence effectors, EsxA counteracts autophagy with various mechanisms. The mutant strain without EsxA fails to inhibit the fusion between autophagosome and lysosome in dendritic cells, implicating EsxA as playing an essential role in autophagic flux [174]. Further study with the ectopically expressed EsxA demonstrates that by upregulating mTOR activity, a negative regulator of autophagy [175], EsxA inhibits autophagy flux and protein degradation in the autophagosome and enhances the intracellular survival of BCG in macrophages [176]. Mass spectrometry analysis shows that both the ectopic expression of EsxA and incubation of the purified EsxA protein upregulate SOD-2 expression in macrophages. Infection of the BCG strain complemented with EsxA also upregulates SOD-2. The knockdown of SOD-2 promotes autophagy and mycobacterial co-localization with lysosomes, resulting in the downregulation of mycobacterial intracellular survival [177]. The protein extract from Mtb culture filtrate also inhibits autophagy, and EsxA purified from extract inhibits autophagy through the upregulation of miR-30a-3p and downregulation of miR-30a-5p. Reverse miRNA transcription recovers autophagy and inhibits mycobacterial intracellular survival, demonstrating the miRNAs’ roles in EsxA inhibition on autophagy [178]. Moreover, when co-expressed with EsxB, EsxA still inhibits autophagy and enhances Mtb intracellular survival [80].

## 3. The Questions that Remain to Be Answered

While EsxA has been extensively studied for the past 20 years, its roles in Mtb pathogenesis and host immune regulation are still not completely understood. Recent studies have aroused more questions that need to be addressed. For instance, although EsxA is a strong T-cell antigen, the high expression profile of EsxA during Mtb infection does not always guarantee an effective Th1 immune response. The well-established role of EsxA MPA in mycobacterial cytosolic translocation is also being challenged, which we believe is much related to genetic knockout of the *esxBA* operon that might have introduced artifacts on the expression or secretion of other co-dependent effectors, resulting in artificial phenotypic changes on mycobacteria.

### 3.1. What Is the Mechanism Underlying the Dual Role of EsxA in Immunoregulation?

As a major antigen, EsxA effectively stimulates Th1 type responses. Combined with proper adjuvants, EsxA induces both strong Th1 and humoral responses and provides comparable protection as the BCG vaccine in animal models [37]. The epitope EsxA_51–70_ induces effective protection against Mtb challenge [179]. When combined with Ag85b, another Mtb immunodominant antigen [180], EsxA vaccination induces even a longer protection effect in mouse lung than BCG (30). The recombinant BCG strain that secretes EsxA provides a much better protection than BCG in animal models [39].

Interestingly, however, EsxA might also contribute to protection failure. Although Mtb antigens specifically induce immune responses, hosts can only limit the infection instead of eradication [181,182], which indicates a possibility that Mtb limits T-cell responses during infection. Later, it has been found that Mtb downregulates and “hides” Ag85b expression during infection, resulting in a significant decrease of activated T cells [183]. Unlike Ag85b, EsxA expression and T-cell recognition are sustained throughout the infection, leading to more differentiated EsxA-specific T cells [184,185,186]. However, the activated T cells fail to secrete cytokines and proliferate, which is probably attributed to exhaustion by constant antigen stimulation [40]. Repeat injections of EsxA subunit vaccine in short intervals cause weaker immune responses and protection, supporting that the constant stimulation of EsxA inhibits host immunity against Mtb [40].

### 3.2. Is Low pH Required for Mycobacterial Cytosolic Translocation?

Biochemical studies have demonstrated that at pH 5 or lower, EsxA undergoes conformational changes and inserts into the liposomal membranes, which is not observed in the EsxA protein from Ms [46,61], indicating that the low pH-dependent MPA is a determinant factor for mycobacterial virulence. The point mutations at Q5 (e.g., Q5K and Q5V) either upregulate or downregulate the low pH-dependent MPA in liposomes and accordingly upregulate or downregulate mycobacterial virulence in cultured macrophages and zebrafish, which has established an unambiguous link between the low pH-dependent MPA and mycobacterial virulence [44]. Most recently, the MPA of EsxA-ST is inhibited by GFP-SC through the specific ST-SC covalent bonding in the liposome assay, and accordingly, the intracellular survival of the Mm(EsxA-ST) is inhibited by the overexpression of GFP-SC in the cultured cells [71]. All the evidence above has established a solid link between the low pH-dependent MPA of EsxA and mycobacterial virulence in infection.

However, numerous studies report that EsxA disrupts lipid membranes at neutral pH. Earlier, either EsxA alone or with EsxB disrupted the lipid bilayer at neutral pH [28]. The purified EsxA or EsxAB exhibited cytolytic activity on human lung epithelial cells and caused cell death [59]. Now, it has become clear that the EsxA-mediated cytolysis at neutral pH is attributed to the residual detergent used in protein purification [53]. The purified EsxA exhibits membrane disruption activity only when it is treated with ASB-14, which is a zwitterionic detergent for endotoxin removal [53]. This protocol had been used in several studies [28,50,187]. Our recent data show that even after several detergent removal procedures, the concentration of the residual ASB-14 in the purified EsxA still ranges from 12 to 32 μg/mL, which is enough to cause cell lysis [188]. In fact, instead of causing cytolysis, the exogenously added EsxA protein without ASB-14 is internalized into the lung epithelial cells and traffics to acidic subcellular compartments, where it inserts into the membranes [188]. These results deny the possibility that EsxA permeabilizes lipid membranes at neutral pH.

Sheep red blood cells (RBCs) have been used to evaluate EsxA or mycobacteria-mediated hemolytic activity. Incubation with the purified EsxA or mycobacteria induces hemolysis, while the deletion of the EsxA or ESX-1 system greatly reduces hemolysis. A transmission electron micrograph shows that Mm disrupts the RBC membranes in a contact-dependent manner [50,53,187,189]. While mycobacteria induce RBC cytolysis in vitro, no report indicates that pathogenic mycobacteria cause tuberculosis by lysing RBC in vivo. Thus, the observed RBC hemolysis might be an in vitro artifact. The RBC membrane consists of ≈40% lipids and 52% of proteins [190,191], which is not equivalent to the membranes used in the liposome and phagosome. Our recent data indicate that the fluidity and net charge of fatty acids have a significant influence on EsxA interaction with the membranes [192]. In the sheep RBC membrane, phosphatidylethanolamine (PE) makes up more than 30% of the membrane phospholipids [193] and confers a much higher membrane fluidity [194]. Thus, it would not be surprising that EsxA had a much higher MPA toward this membrane, even at neutral pH [192]. Considering that proteins make up around 52% of the RBC membrane and mycobacteria-mediated hemolysis depends on direct contact with RBC [190], the possibility that mycobacteria interact with RBC membrane proteins to induce membrane rupture cannot be overlooked.

A recent study has shown that mycobacteria are still able to escape from the phagosome in the presence of bafilomycin, which is a reagent inhibiting acidification [53]. However, there are three major concerns regarding the data. One, while the lysotracker is generally used as an intracellular pH indicator, it is not for accurate and quantitative pH measurement. It is possible that acidification was partially inhibited, but the pH was still low enough for EsxA to permeabilize phagosomes. Second, in the report, the rate of phagosome permeabilization for MmWT was only ≈6%, which only represents a small population of the bacteria. Third, bafilomycin appeared to enhance the permeabilization rate of MmΔRD1 (from 0.82% to 3.2%), but not MmWT, suggesting that bafilomycin treatment could generate unexpected artifacts to mislead the results. In fact, it has been reported that the treatment of bafilomycin not only inhibits phagosome acidification, but it also alters phagosome membrane composition [195], which is critical for the function of intracellular pathogen-secreted effectors [196,197]. Given that the EsxA MPA is also affected by membrane composition [192], it is possible that bafilomycin treatment affects the membrane composition, which allows mycobacteria to escape from the phagosome even when acidification is inhibited.

### 3.3. Is EsxA Required for Mycobacterial Cytosolic Translocation?

Genetic studies using the strains with the deletion or disruption of *esxA* or *esxBA* have shown that EsxA is required for mycobacterial virulence [44,50,53,77,86]. However, a recent study has shown that the transposon mutants, which are defected in EsxAB secretion, are still able to escape from the phagosome, indicating that factors other than EsxA are required for mycobacterial cytosolic translocation and virulence [43]. The major concern for this report is that the gene deletion caused by transposon insertion might induce a genetic compensatory mechanism in mycobacteria and produce artifacts. Moreover, it has been shown that the expression and secretion of EsxAB as well as numerous factors encoded within or outside the ESX-1 locus are co-dependent on each other [42,43,54,55,75,198,199,200]. For instance, the deletion of *esxA* blocks the secretion of EspA in Mtb [75]. In Mm, the deletion of *esxA* or *esxBA* reduces the expression and secretion of EspB and EspF as well as EsxN encoded within the ESX-5 locus, but it upregulates the secretion of MMAR_2929 from the Sec system [54]. Moreover, the disruption of other ESX-1 genes alters the expression or secretion of EsxA. The deletion of *espE* or *espF* significantly upregulates the expression and secretion of EsxA but downregulates hemolysis [55]. The co-dependency among EsxAB and various effectors is summarized in Table 1 [54,55,75], which greatly increases the complexity of dissecting the roles of each individual factor in mycobacterial pathogenesis. Considering the co-dependency complexity, the results obtained by gene deletion and disruption need to be carefully interpreted.

Three lines of evidence obtained by the approaches other than gene deletion strongly support the essential role of EsxA MPA in mycobacterial cytosolic translocation. First, the point mutations at the residue Q5, which do not affect the expression and secretion of EsxA, hence presumably do not affect other co-dependent factors, upregulate or downregulate the EsxA MPA, and accordingly upregulate or downregulate the mycobacterial cytosolic translocation and virulence [44]. Second, an inducible knockdown of EsxAB at the post-translational level through a DAS4 degradation tag that is engineered to the C-terminus of EsxB results in attenuated mycobacterial virulence [71]. Third, the Mm(EsxA-ST) strain has a normal expression and secretion of EsxA-ST, but its virulence is attenuated by the overexpression of GFP-SC in the infected host cells, suggesting that EsxA-ST MPA is inhibited by GFP-SC at the post-secretion level, where other co-dependent factors are not likely to be affected [71].

## 4. Future Directions

Current studies suggest that EsxA is necessary but not sufficient for mycobacterial phagosome rupture and cytosolic translocation solely; other factors may also contribute to this process. It has been reported that EsxA works with phthiocerol dimycocerosates (PDIM) to mediate phagosome rupture [201,202], and PDIM alone can cause a certain level of hemolysis with Mm [203]. The disruption of other ESX-1 substrates also impacts mycobacterial phagosome escape [43,53]. A recent study has reported that the deletion of *espE/F* significantly inhibits Mm-mediated hemolysis, while the expression and secretion of EsxAB were not affected [55], indicating that EsxAB is not sufficient for mycobacterial virulence. Future studies on the other factors that are involved in mycobacterial cytosolic translocation are needed.

An earlier study has shown that the Mtb without ESX-1 locus exhibits a stronger association with lung epithelial cells, indicating that EsxA plays a role in cell invasion [28]. The Mm strain with the deletion of *esxBA* has an increased adherence to the murine macrophages [44]. Most recently, our preliminary data have shown that the Mm strain with EsxA deletion has an increased adherence but a decreased invasion in WI-26 [188]. Recent studies have shown that the secreted EsxAB remains associated with the mycobacterial cell wall, rather than released into the medium, and the cell wall-associated EsxAB correlates with bacterial virulence [85,198,204]. Thus, it is possible that EsxAB mediates the mycobacterial adherence and invasion into host cells, which implicates a new role of EsxAB in mycobacterial pathogenesis.

Upon acidification, EsxA inserts into the liposomal membrane and forms a transmembrane structure [61], and the exogenously added EsxA with a NBD label is internalized into the lung epithelial cells and inserts into the host membranes within the acidic subcellular compartment [188]. Most recently, molecular dynamic simulation shows that EsxA inserts into lipid membranes in the form of a C4 oligomer [205]. Thus, current studies support that EsxA forms a pore or channel across the lipid membrane. However, the structure of the putative EsxA pore is still not available; hence, its exact function is unknown, which warrants further investigations in the future.

Current studies have shown that EsxA, but not EsxB, possesses the acidic pH-dependent MPA, which is essential for phagosome rupture [46,67]. EsxA alone has a Tm at 37 °C and the heterodimer has a Tm at 55 °C, suggesting that EsxB stabilizes EsxA at 37 °C [206]. EsxA and EsxB are secreted through the ESX-1 system as a heterodimer [75], and the C-terminal YxxD/E motif of EsxB is required for the secretion [207]. The evidence above supports that EsxB functions as a chaperone of EsxA for its secretion and stabilization. However, in addition to functioning as a chaperon, EsxB has been shown to be responsible for triggering Ca^2+^ response in human neutrophils in a pertussis toxin-sensitive manner, suggesting that EsxB contributes specifically to neutrophil recruitment and activation during Mtb infection [208]. Therefore, for a more complete picture of ESX-1 effectors’ roles, the roles of EsxB need more investigation. Moreover, due to the mutual codependence among ESX-1 effectors, a study of EsxB without having impact on other ESX-1 effectors needs to be carefully considered.

## Figures and Tables

**Table 1:** Expression and secretion co-dependence among ESX-1 effectors.

Effect of EsxA’s Deletion on Other Effectors			Effect of Other Effectors’s Deletion on EsxA		
ΔEsxA (48)	CL ^a^	CF ^b^	EsxA	CL ^a^	CF ^b^
EsxB	↓	↓	ΔEsxB [54]	↓	↓
EspB	↓	↓	ΔEspB [54]	↓	↓
EspF	↓	↓	ΔEspE [55]	↑	↑
EspK	↓	↓	ΔEspF [55]	↑	↑
EspJ	↑	↓	ΔEspK [54]	-	↓
PPE68	↓	↓	ΔEspJ [54]	-	↓
			ΔEspI [43]	↓	↓
			ΔEspG1 [43]	↓	↓
			ΔEspH [43]	↓	↓

^a^: cell lysate; ^b^: culture filtrate; **↓**: downregulation; **↑**: upregulation; -: unaffected.
